# Supplementary material for: Leveraging multi-way interactions for systematic prediction of pre-clinical drug combination effects
Source: Nat Commun. 2020 Dec 1;11:6136. doi: 10.1038/s41467-020-19950-z (PMC7708835; doi:10.1038/s41467-020-19950-z)
Supplement: Supplementary file 1 — Supplementary Information [file 41467_2020_19950_MOESM1_ESM.pdf]

Supplementary information: Leveraging multi-way interactions for systematic prediction of pre-clinical drug combination effects

Heli Julkunen[1], Anna Cichonska[1,2,3], Prson Gautam[3], Sandor Szedmak[1], Jane Douat[1], Tapio Pahikkala[2], Tero Aittokallio[1,3,4,5,6,*], and Juho Rousu[1,*]

[1]Department of Computer Science, Helsinki Institute for Information Technology HIIT, Aalto University, Espoo, Finland
[2]Department of Future Technologies, University of Turku, Turku, Finland
[3]Institute for Molecular Medicine Finland FIMM, University of Helsinki, Helsinki, Finland
[4]Department of Mathematics and Statistics, University of Turku, Turku, Finland
[5]Department of Cancer Genetics, Institute for Cancer Research, Oslo University Hospital, Oslo, Norway
[6]Oslo Centre for Biostatistics and Epidemiology, University of Oslo, Oslo, Norway
[*]Corresponding author. juho.rousu@aalto.fi, tero.aittokallio@helsinki.fi

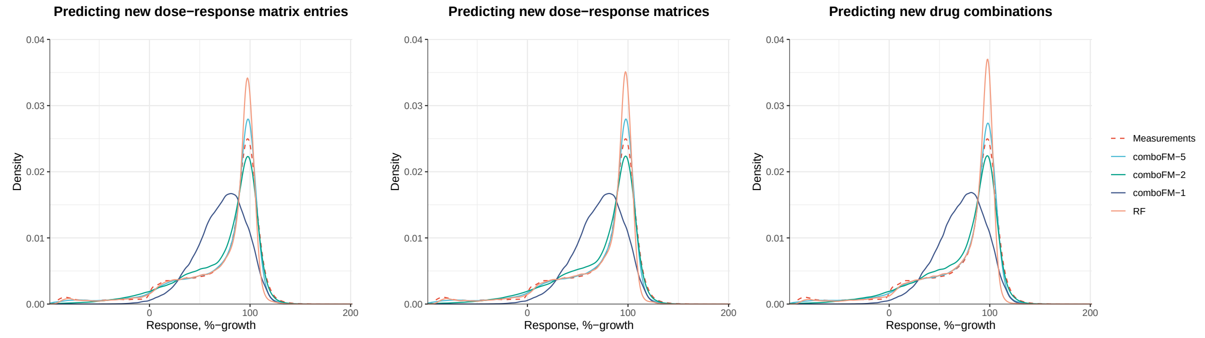

Figure 1: Distributions of the measured drug-dose combination responses (dashed red curve) and those predicted by 5th (comboFM-5, light blue line), 2nd (comboFM-2, turquoise line) and 1st order comboFM (comboFM-1, dark blue line) and random forest (RF, solid peach line), measured by percentage growth of the cancer cell lines, in the selected subset of the NCI-ALMANAC dataset.

a

Tissue	Predicting new dose-response matrix entries comboFM-2	comboFM-1	RF	Predicting new dose-response matrices comboFM-2	comboFM-1	RF	Predicting new drug combinations comboFM-2	comboFM-1	RF
Renal	1.00e+00	**< 1.00e-05**	1.00e+00	1.00e+00	**< 1.00e-05**	1.00e+00	1.00e+00	**< 1.00e-05**	**< 1.00e-05**
NSC Lung	**< 1.00e-05**	**< 1.00e-05**	**< 1.00e-05**	**< 1.00e-05**	**< 1.00e-05**	1.00e+00	1.00e+00	**< 1.00e-05**	**2.12e-02**
Haematological	**< 1.00e-05**	2.40e-01	**< 1.00e-05**	**3.81e-02**	**6.91e-04**	**< 1.00e-05**	**< 1.00e-05**	6.26e-02	1.00e+00
Colon	**< 1.00e-05**	**< 1.00e-05**	**< 1.00e-05**	**1.76e-02**	**< 1.00e-05**	**4.20e-04**	1.67e-01	**< 1.00e-05**	7.24e-01
Prostate	1.00e+00	**< 1.00e-05**	**< 1.00e-05**	1.00e+00	**< 1.00e-05**	1.00e+00	1.00e+00	**< 1.00e-05**	**2.55e-05**
Ovarian	**5.04e-03**	**< 1.00e-05**	**< 1.00e-05**	1.00e+00	**< 1.00e-05**	**1.27e-05**	2.32e-01	**< 1.00e-05**	**< 1.00e-05**
Melanoma	1.00e+00	**< 1.00e-05**	**< 1.00e-05**	**6.64e-03**	**< 1.00e-05**	**< 1.00e-05**	**< 1.00e-05**	**< 1.00e-05**	**< 1.00e-05**
Breast	**2.11e-04**	**< 1.00e-05**	**1.50e-02**	**< 1.00e-05**	**< 1.00e-05**	**< 1.00e-05**	**1.06e-05**	**< 1.00e-05**	1.00e+00
CNS	1.00e+00	**< 1.00e-05**	**< 1.00e-05**	**3.57e-03**	**< 1.00e-05**	5.17e-02	**< 1.00e-05**	**< 1.00e-05**	**< 1.00e-05**

b

Drug classes	Predicting new dose-response matrix entries comboFM-2	comboFM-1	RF	Predicting new dose-response matrices comboFM-2	comboFM-1	RF	Predicting new drug combinations comboFM-2	comboFM-1	RF
Chemo - Chemo	**1.64e-02**	**< 1.00e-05**	**< 1.00e-05**	**< 1.00e-05**	**< 1.00e-05**	**< 1.00e-05**	**3.82e-02**	**< 1.00e-05**	**< 1.00e-05**
Chemo - Other	7.25e-02	**< 1.00e-05**	**< 1.00e-05**	**< 1.00e-05**	**< 1.00e-05**	**3.97e-02**	**< 1.00e-05**	**< 1.00e-05**	**< 1.00e-05**
Targeted - Chemo	1.00e+00	**< 1.00e-05**	**< 1.00e-05**	1.00e+00	**< 1.00e-05**	1.00e+00	**< 1.00e-05**	**< 1.00e-05**	**< 1.00e-05**
Targeted - Other	**< 1.00e-05**	**< 1.00e-05**	**4.69e-03**	**< 1.00e-05**	**< 1.00e-05**	1.00e+00	**< 1.00e-05**	**< 1.00e-05**	**< 1.00e-05**
Targeted - Targeted	**< 1.00e-05**	**< 1.00e-05**	**< 1.00e-05**	**2.16e-03**	**< 1.00e-05**	1.00e+00	**< 1.00e-05**	**< 1.00e-05**	**1.60e-04**
Other - Other	**< 1.00e-05**	**< 1.00e-05**	1.00e+00	**< 1.00e-05**	**< 1.00e-05**	**1.64e-02**	**< 1.00e-05**	**< 1.00e-05**	**< 1.00e-05**

Table 1: Bonferroni corrected p-values from two-sided paired Wilcoxon signed rank sum test between the predictions of 5th-order comboFM (comboFM-5) and the compared models, 1st order (comboFM-1) and 2nd order (comboFM-2) models, as well as random forest (RF) in each tissue type and drug class (135 independent tests; p-values above 1 after multiple testing correction are set to 1 and statistically significant ($\alpha = 0.05$) p-values are marked in bold).

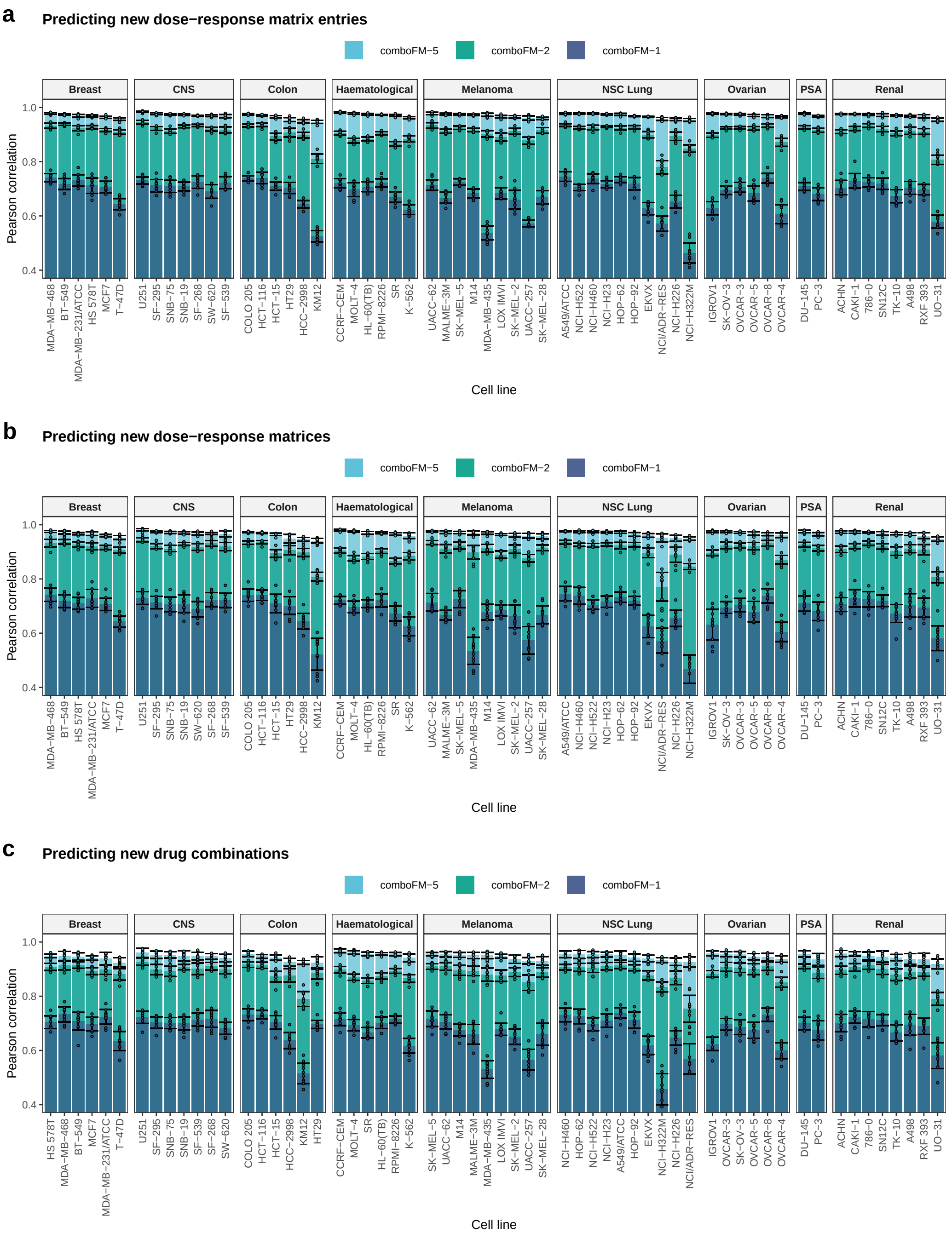

Figure 2: Effect of increasing order of modeled feature interactions in 1st (comboFM-1), 2nd (comboFM-2) and 5th order comboFM (comboFM-5) across the NCI-60 panel of cell lines and tissue types in terms of Pearson correlation in the three prediction scenarios: (a) predicting new dose-response matrix entries, (b) predicting new dose-response matrices and (c) predicting new drug combinations. The dots show the Pearson correlations between the measured and predicted responses computed separately for each outer fold of the nested cross-validation ($n = 10$), the bar denotes the mean over the folds and the error bars show the standard deviation over the folds.

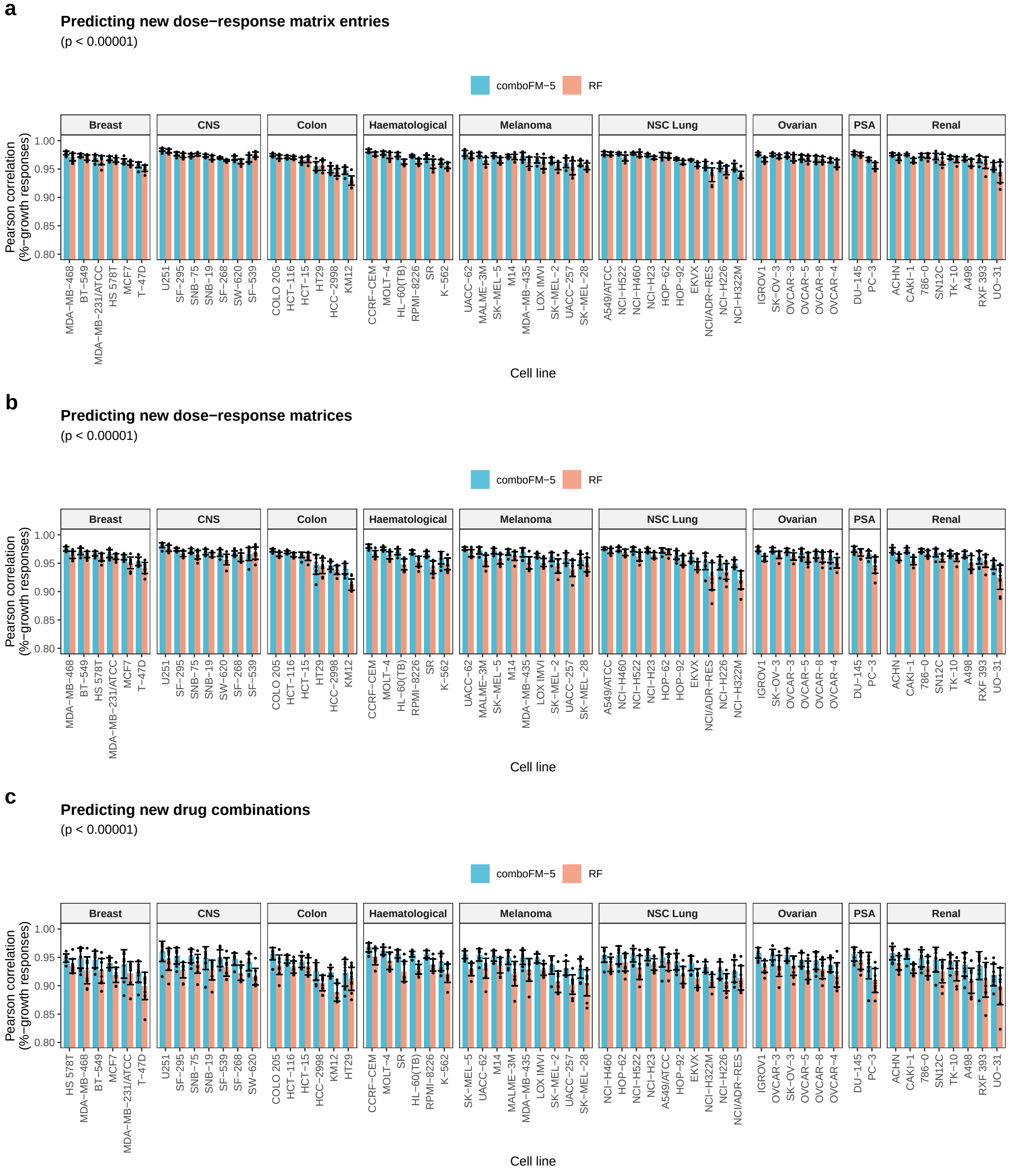

Figure 3: Performance of 5th order comboFM (comboFM-5) and random forest (RF) across the NCI-60 panel of cell lines and tissue types in terms of Pearson correlation between the measured and predicted responses, in the three prediction scenarios: (a) predicting new dose-response matrix entries, (b) predicting new dose-response matrices and (c) predicting new drug combinations. The dots show the Pearson correlations computed separately for each outer fold of the nested cross-validation ($n = 10$), the bar denotes the mean over the folds and the error bars show the standard deviation over the folds. The p-values in each setting demonstrate the improved prediction accuracy of comboFM-5, as assessed with one-sided Wilcoxon signed-rank test.

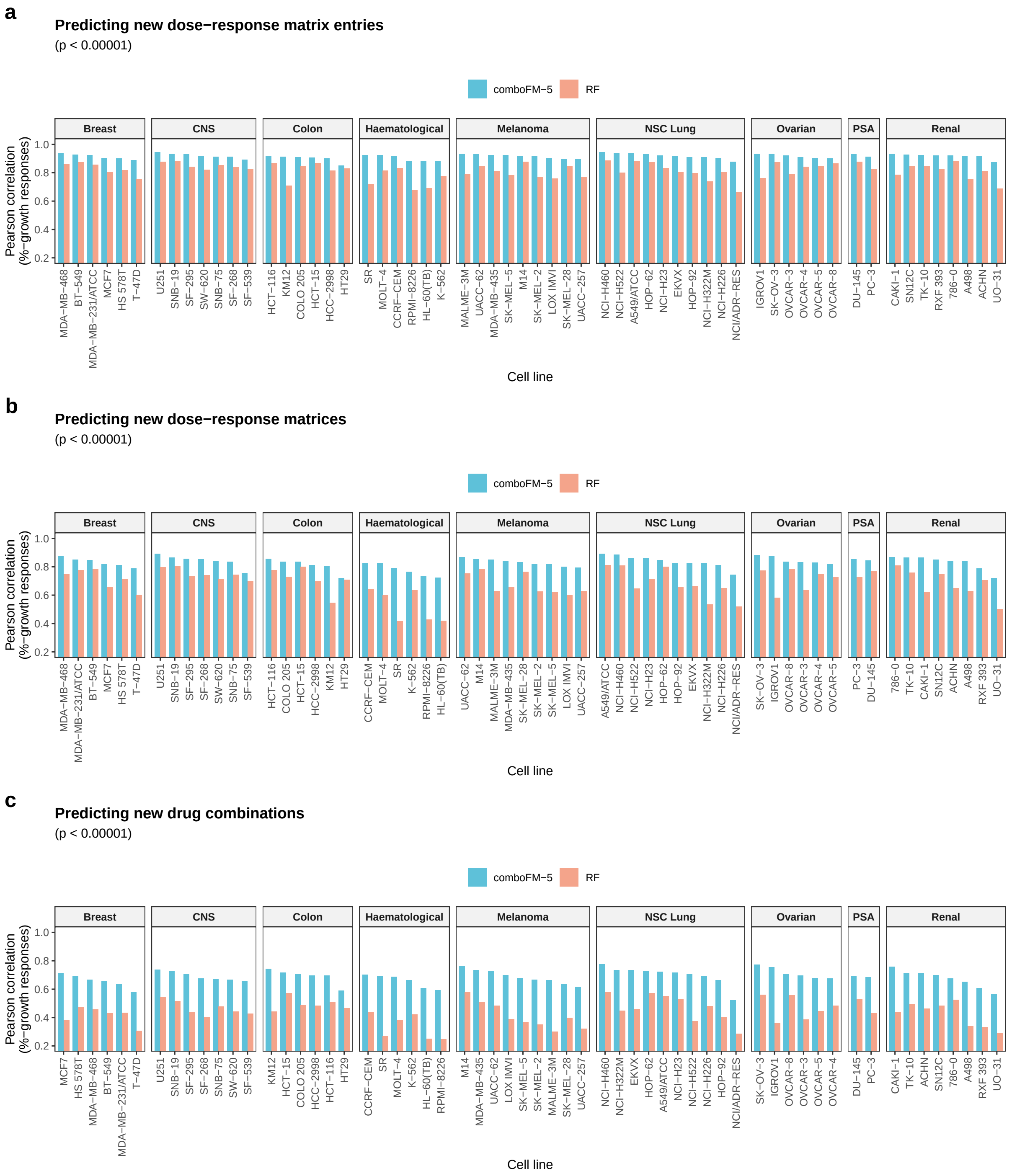

Figure 4: Performance of 5th order comboFM (comboFM-5) and random forest (RF) across the NCI-60 panel of cell lines and tissue types in terms of Pearson correlation between the measured and predicted ComboScores, computed over complete predicted dose-response matrices, in the three prediction scenarios: (a) predicting new dose-response matrix entries, (b) predicting new dose-response matrices and (c) predicting new drug combinations. The p-values in each setting demonstrate the improved prediction accuracy of comboFM-5, as assessed with one-sided Wilcoxon signed-rank test.

	comboFM-5		comboFM-2		comboFM-1		RF
	CPU	GPU	CPU	GPU	CPU	GPU	CPU
Model training (min)	534.386	43.192	61.207	22.179	7.745	19.006	1450.780
Predicting with a trained model (min)	0.368	0.009	0.037	0.005	0.005	0.004	1.535

Table 2: Training and prediction times of 1st (comboFM-1), 2nd (comboFM-2), and 5th order comboFM (comboFM-5) compared to random forest (RF) with optimal parameters for one round of outer 10-fold CV. comboFM models were trained for 200 epochs with batch size of 1024, using Adam optimizer with a learning rate of 0.001. random forest model was trained using 512 trees and considering all features at each split. As comboFM can be effectively trained using graphical processing units (GPU), the training times were recorded using both GPUs and CPUs for comparison with random forest. All CPU experiments were performed on an 2x12 core Intel Xeon E5 2680 v3 2.50GHz CPU with 128 GB memory. The GPU experiments were run on a 32 GB NVIDIA Tesla V100 GPU.

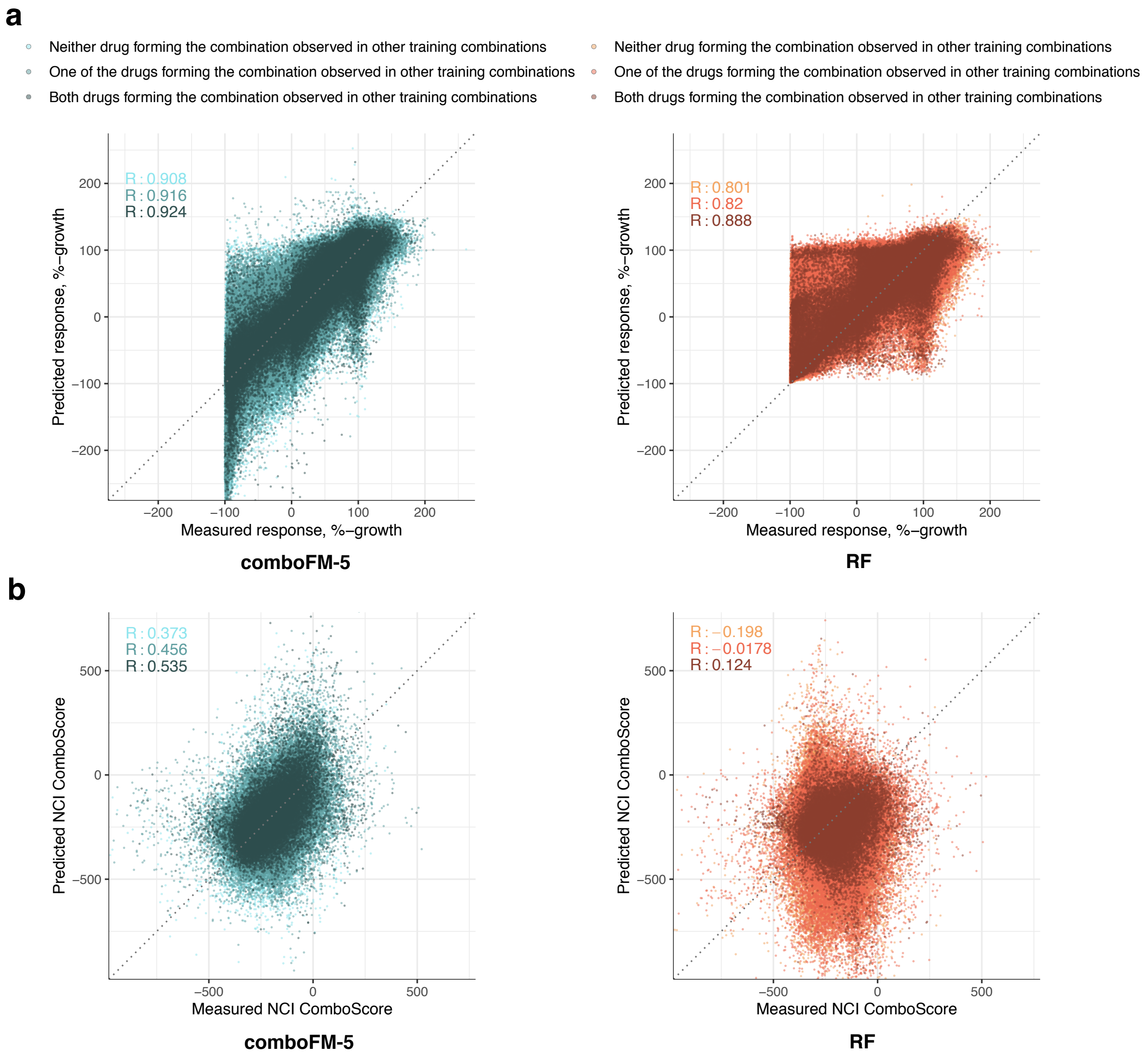

Figure 5: Predictive performance of 5th order comboFM (comboFM-5) and random forest (RF) in a validation set consisting of the NCI-ALMANAC data not used in the method development and cross-validation, where both of the models were trained using the full development set and monotherapy responses of single drugs in the validation set (476 distinct monotherapy dose-responses). a) Scatter plots between the measured and predicted dose-responses, stratified according to the number of drugs in each combination observed in other combinations of the training set (color coding). b) Scatter plots between measured and predicted NCI ComboScores computed based on the model-predicted dose-response matrices.

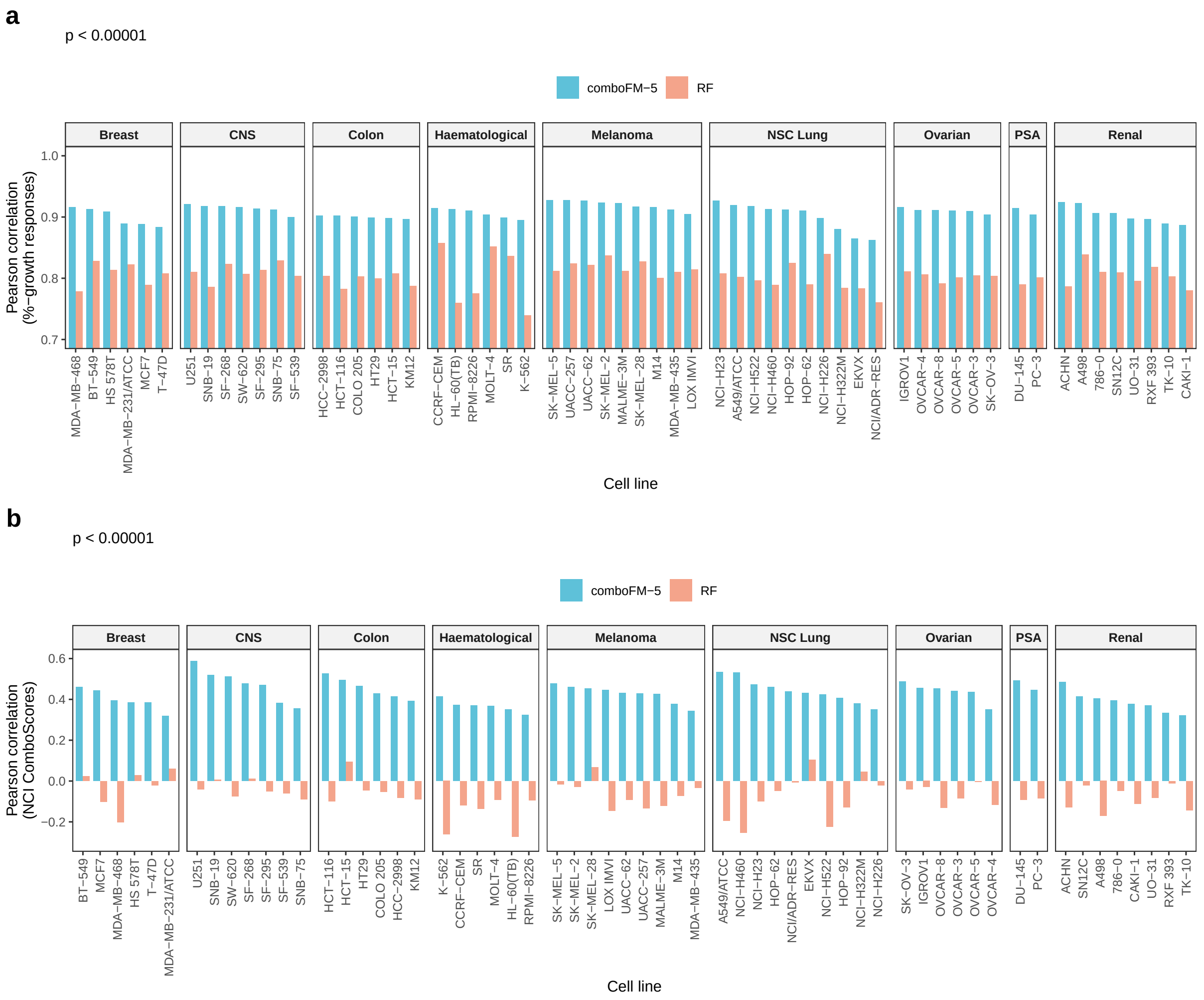

Figure 6: Predictive performance of 5th order comboFM (comboFM-5) and random forest (RF) across the NCI-60 panel of cell lines and tissue types, in terms of Pearson correlation (R) between measured and predicted a) dose-responses and b) NCI ComboScores, in a validation set consisting of the NCI-ALMANAC data not used in the method development and cross-validation, where both of the models were trained using the full development set and the monotherapy responses of single drugs in the validation set. The p-values in each setting demonstrate the improved prediction accuracy of comboFM-5, as assessed with one-sided Wilcoxon signed-rank test.

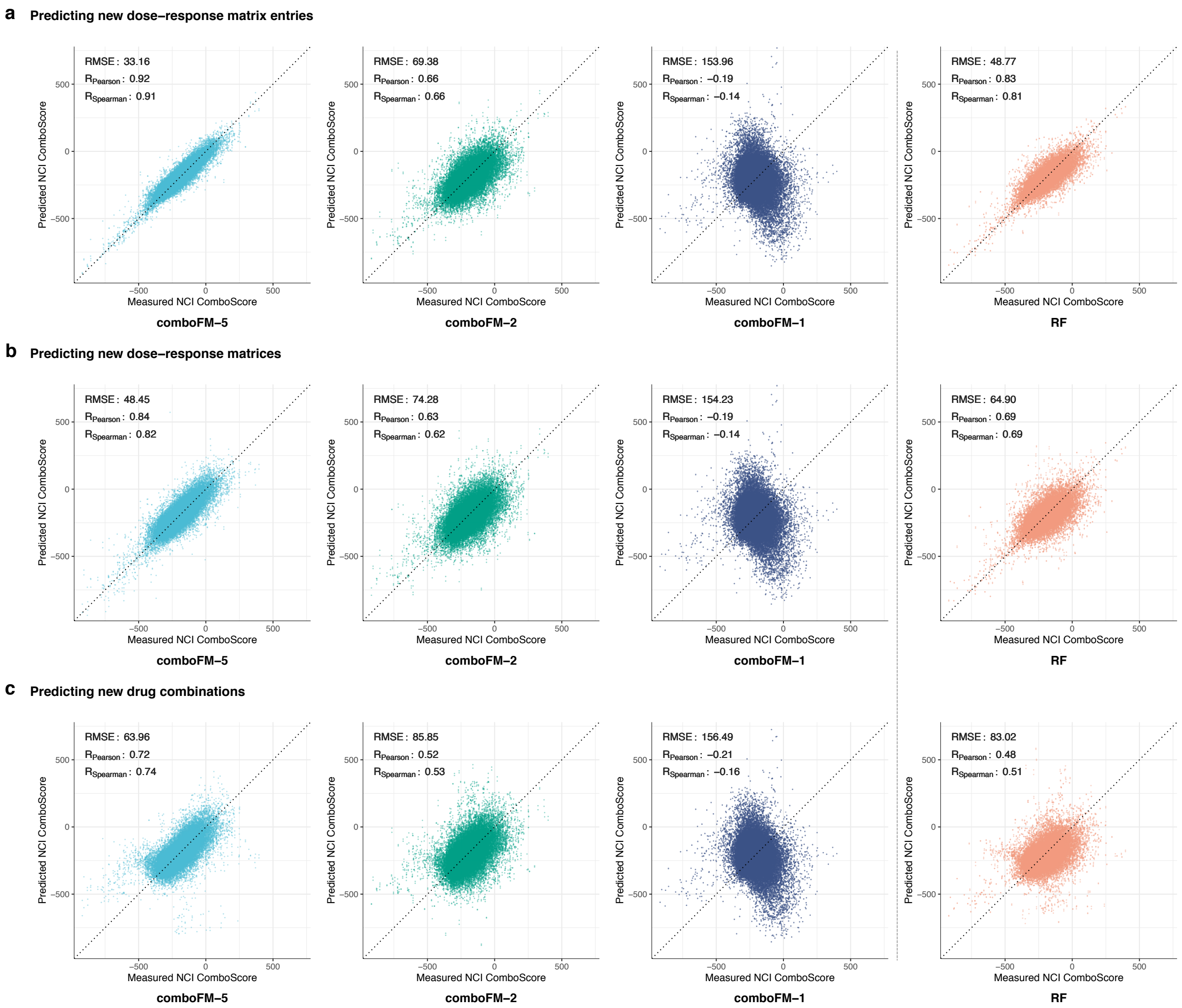

Figure 7: Predictive performance of 5th (comboFM-5, 2nd (comboFM-2) and 1st order (comboFM-1) comboFM and random forest (RF) in quantifying drug combination synergy scores, measured by NCI ComboScore computed over complete predicted dose-response matrices, as scatter plots between the measured and predicted synergy scores in the three prediction scenarios:(a) predicting new dose-response matrix entries, (b) predicting new dose-response matrices and (c) predicting new drug combinations. Root mean squared error (RMSE), Pearson correlation (R_{Pearson}) and Spearman correlation (R_{Spearman}) are reported as averages over all computed NCI ComboScores.

a

Prediction scenario	$R_{Pearson}$	$R_{Spearman}$	RMSE	AUC
Predicting new dose-response matrix entries	0.92	0.91	33.16	0.98
Predicting new dose-response matrices	0.84	0.82	48.45	0.95
Predicting new drug combinations	0.72	0.74	63.96	0.91

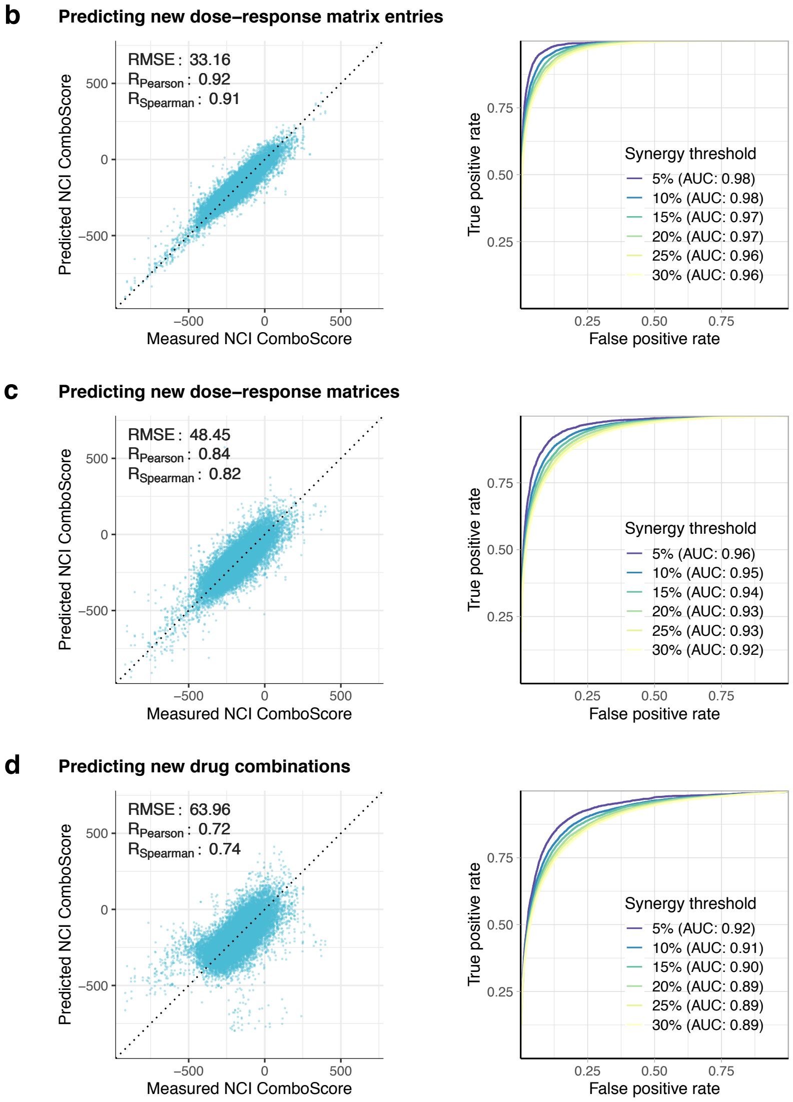

Figure 8: (a) Summary of the average Pearson ($R_{Pearson}$) and Spearman ($R_{Spearman}$) correlations, root mean squared error (RMSE) and area under the receiver operating characteristics curve (AUC) of 5th order comboFM in quantifying drug combination synergy scores, measured by NCI ComboScore computed over compelete predicted dose-response matrices in the three prediction scenarios. The AUC is computed based on considering 10% of combinations with the highest NCI ComboScore values as synergistic. (b-d) Predictive performance of 5th order comboFM as scatter plots between the original and predicted NCI ComboScores and receiver operating characteristic (ROC) curves to evaluate the model performance in classifying drug combinations as synergistic vs. non-synergistic with varying thresholds for synergy, in the three prediction scenarios: (b) predicting new dose-response matrix entries, (c) predicting new dose-response matrices and (d) predicting new drug combinations. The performance metrics are reported as an average over all computed synergy scores. The classification was repeated with 6 different thresholds: drug combinations with a ranked ComboScore in the top [5%, 10%, . . . , 30%] were labeled as synergistic. Area under the ROC curve (AUC) is shown in parenthesis.

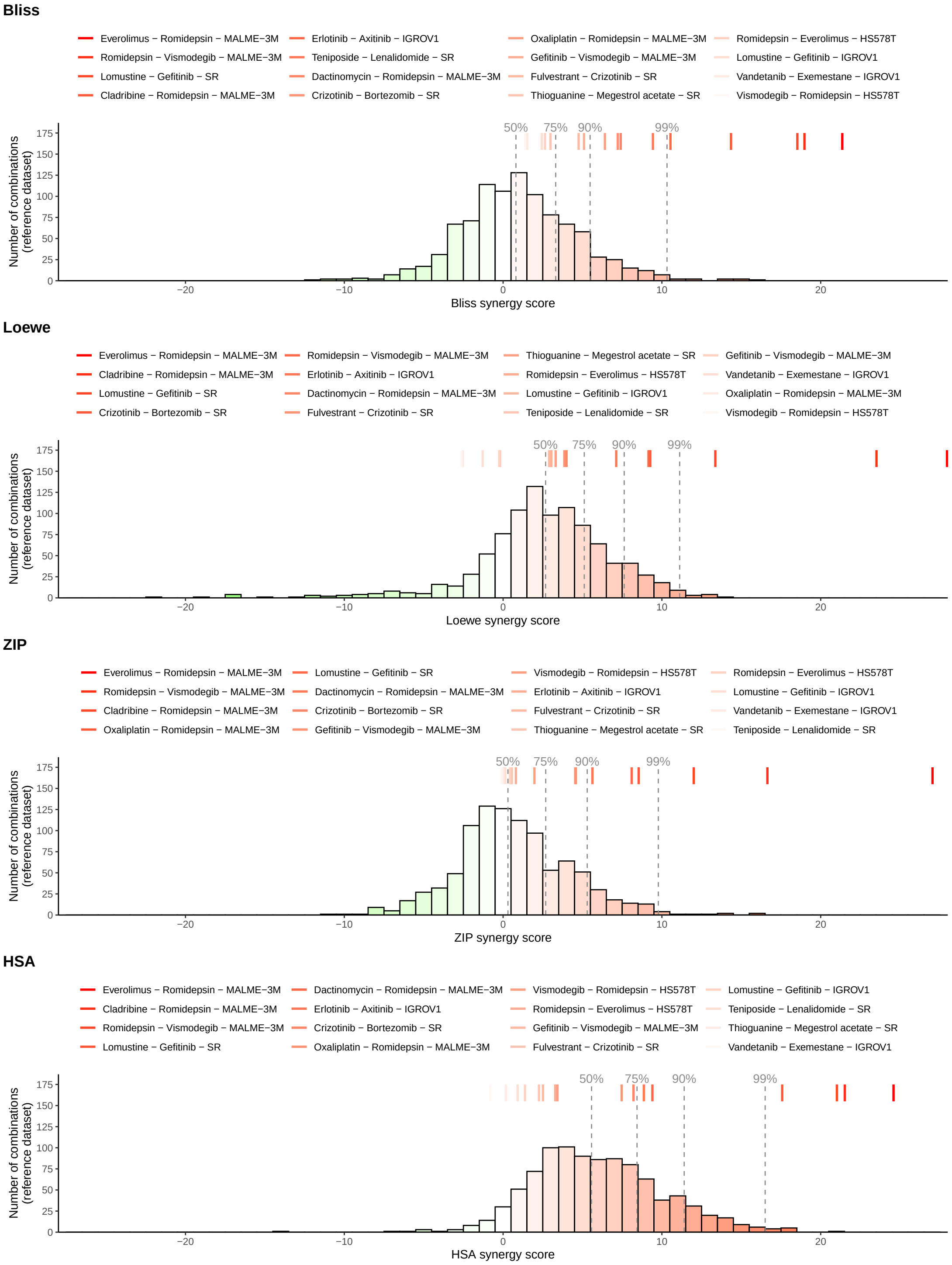

Figure 9: Measured drug combination synergy scores in the experimental validation, evaluated using four synergy models: zero interaction potency (ZIP), Bliss, Loewe, and highest single agent (HSA) models. In-house experimental validation of 16 selected predictions in specific cell lines are shown as colored lines (on top), and the histogram shows a background distribution from in-house reference dataset that comprises of 60 drug combinations tested against 16 KRAS-mutants pancreatic ductal adenocarcinoma cell lines (unpublished data, see Methods). The scores are reported for the most synergistic 2×2 area of the dose-response matrix. The color scale corresponds to the synergy scores (green - antagonistic response, white - independent response, red - synergistic response). Dashed lines denote the percentiles of the background distribution obtained using the same experimental setup.

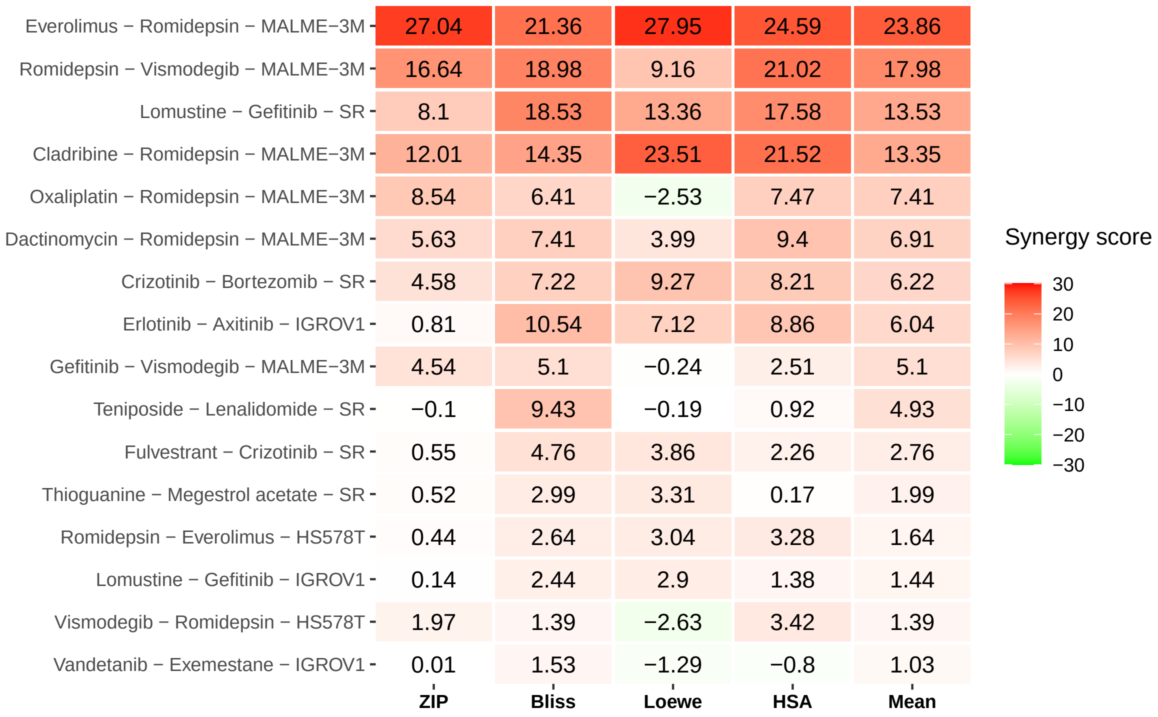

Figure 10: Observed drug combination synergies from experimental validation. For each combination, the synergies were evaluated using four synergy models: zero interaction potency (ZIP), Bliss, Loewe and highest single agent (HSA) models, and the scores for the most synergistic concentration area of the dose-response matrix are reported. Color scale indicates the synergy score (green - antagonistic response, white - independent response, red - synergistic response).

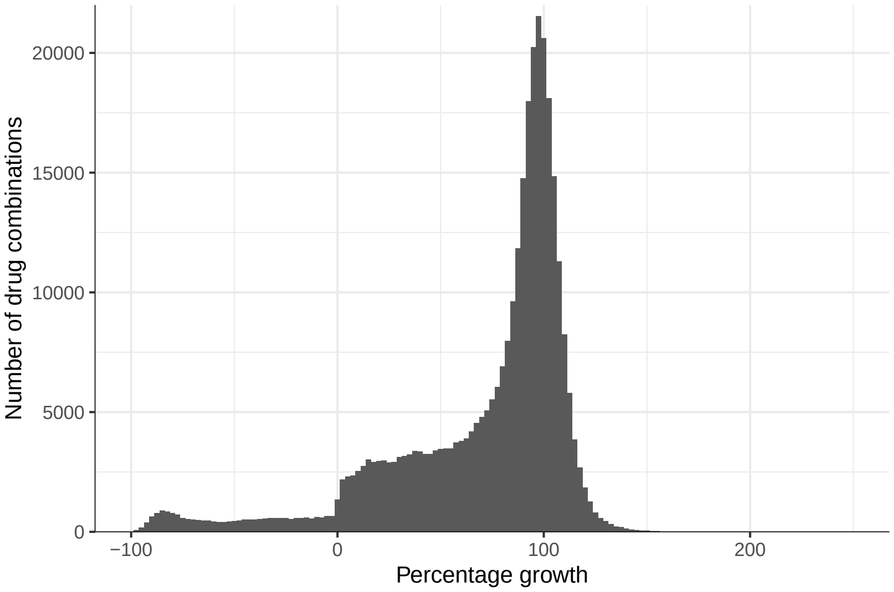

Figure 11: Distribution of the drug-dose combination responses, measured by percentage growth of the cancer cell lines, in the selected subset of the NCI-ALMANAC dataset.

Drug name	Mechanism/targets	Class
2-Fluoro Ara-A	Antimetabolite; Purine analog	Chemotherapy
Amifostine	Antineoplastic detoxifying agent	Chemotherapy
Arsenic trioxide	Antineoplastic agent	Chemotherapy
Axitinib	VEGFR, PDGFR, KIT inhibitor	Targeted therapy
Bortezomib	Proteasome inhibitor (26S subunit)	Chemotherapy
Cabazitaxel	Taxane microtubule stabilizer, antimitotic	Chemotherapy
Carmustine	Alkylating agent	Chemotherapy
Chlorambucil	Nitrogen mustard alkylating agent	Chemotherapy
Cladribine	Antimetabolite; Purine analog	Chemotherapy
Crizotinib	ALK, c-Met inhibitor	Targeted therapy
Cytarabine hydrochloride	Antimetabolite, interferes with DNA synthesis	Chemotherapy
Dacarbazine	Alkylating agent	Chemotherapy
Dactinomycin	RNA and DNA synthesis inhibitor	Chemotherapy
Daunorubicin hydrochloride	Topoisomerase II inhibitor	Chemotherapy
Erlotinib hydrochloride	EGFR inhibitor	Targeted therapy
Estramustine phosphate sodium	Alkylating agent	Chemotherapy
Everolimus	binds FKBP12, causes inhibition of mTORC1	Targeted therapy
Exemestane	Aromatase inhibitor	Other
Floxuridine	Antimetabolite	Chemotherapy
Fulvestrant	Estrogen receptor antagonist	Other
Gefitinib	EGFR inhibitor	Targeted therapy
Hydroxyurea	Antineoplastic agent	Chemotherapy
Ifosfamide	Nitrogen mustard alkylating agent	Chemotherapy
Lenalidomide	Immunomodulatory	Other
Lomustine	Alkylating nitrosourea compound	Chemotherapy
Megestrol acetate	Progestogen	Other
Melphalan	Nitrogen mustard alkylating agent	Chemotherapy
Methoxsalen	Antineoplastic agent	Chemotherapy
Mitotane	Antineoplastic agent	Chemotherapy
Mitoxantrone	Topoisomerase II inhibitor	Chemotherapy
Oxaliplatin	Platinum-based antineoplastic agent	Chemotherapy
Pemetrexed Disodium	Dihydrofolate reductase inhibitor	Chemotherapy
Pralatrexate	Antifolate	Chemotherapy
Procarbazine hydrochloride	Alkylating agent	Chemotherapy
Quinacrine hydrochloride	Antineoplastic agent	Chemotherapy
Romidepsin	HDAC inhibitor	Other
Ruxolitinib	JAK1&2 inhibitor	Targeted therapy
Sorafenib tosylate	B-Raf, FGFR-1, VEGFR-2 & -3, PDGFR-beta, KIT, and FLT3 inhibitor	Targeted therapy
Tamoxifen citrate	Estrogen receptor antagonist	Other
Teniposide	Topoisomerase II inhibitor	Chemotherapy
Thalidomide	Immunosuppresant	Other
Thioguanine	Antimetabolite; Purine analog	Chemotherapy
Thiotepa	Alkylating agent	Chemotherapy
Uracil mustard	Alkylating agent	Chemotherapy
Valrubicin	Topoisomerase II inhibitor	Chemotherapy
Vandetanib	VEGFR,EGFR, RET inhibitor	Targeted therapy
Vinblastine sulfate	Mitotic inhibitor. Vinca alkaloid microtubule depolymerizer	Chemotherapy
Vincristine sulfate	Mitotic inhibitor. Vinca alkaloid microtubule depolymerizer	Chemotherapy
Vinorelbine tartrate	Mitotic inhibitor. Vinca alkaloid microtubule depolymerizer	Chemotherapy
Vismodegib	Smothened (Hh) inhibitor	Targeted therapy

Table 3: List of drug compounds in the selected subset of the NCI-ALMANAC dataset.

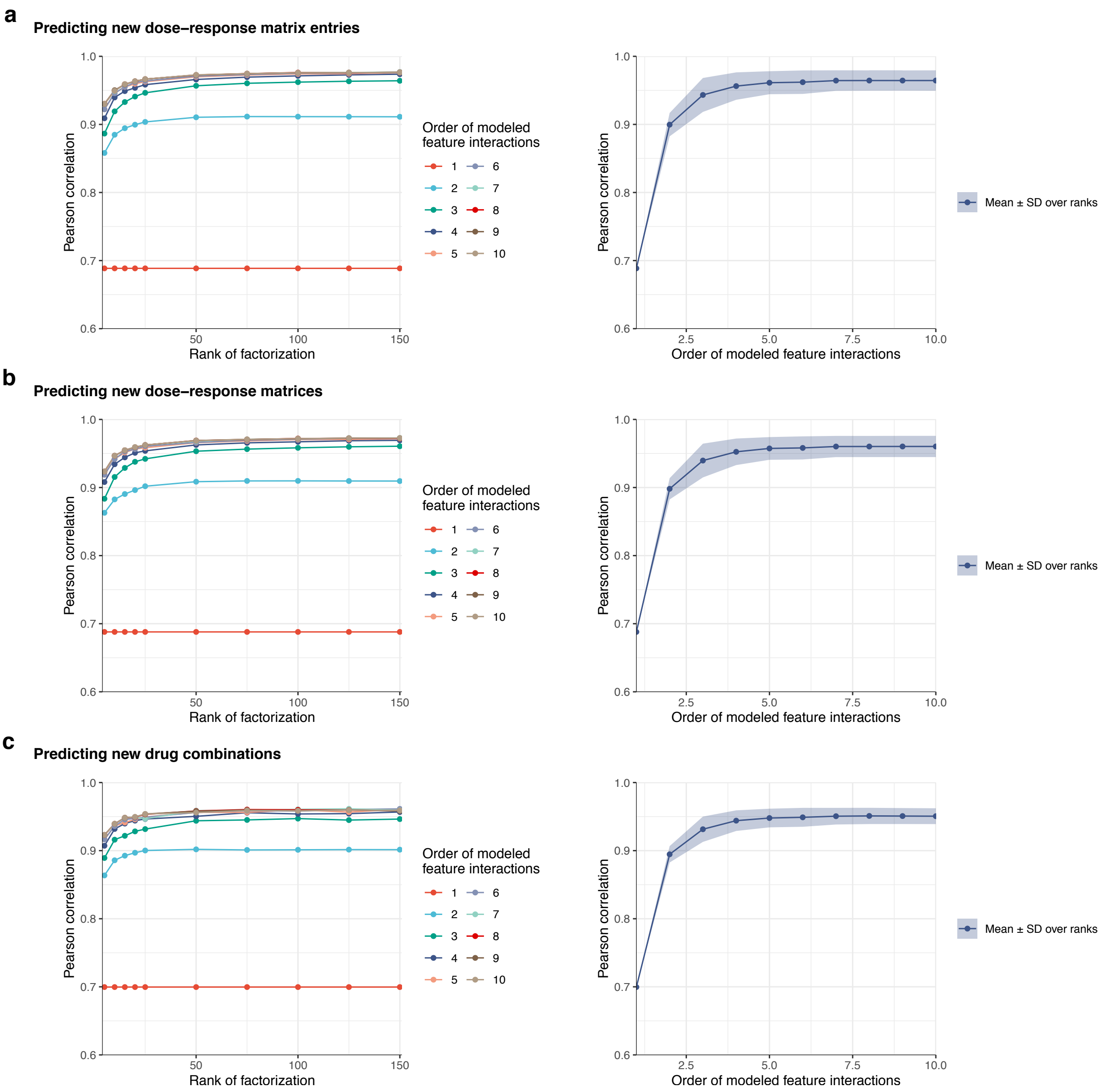

Figure 12: Impact of the rank of the factorization (left pane) on the predictive performance for different orders of the factorization machine ($\{1,2,...,10\}$), measured by Pearson correlation, in the first outer test fold in the three scenarios of predicting (a) new dose-response matrix entries, (b) new dose-response matrices and (c) new drug combinations. Impact of the order of the factorization machine, i.e. order of the model feature interactions (right pane), on the predictive performance, measured by Pearson correlation, in the first outer test fold in the three scenarios, with the mean and standard deviation computed over different ranks in the range $\{5, 10, 15,...,25, 50, 75..., 150\}$.

a

Cell line	Histologic Type	Source	Species	Source code	Growth Medium	Cell seeding amount /well (384-well plate)
Hs-578T	breast carcinoma	ATCC	human	ATCC® HTB-126™	RPMI 1640+ 10%FBS+ 2mM Glutamine+ 1% Pen/Strep	1000
MALME-3M	melanoma	ATCC	human	ATCC® HTB-64™	IMDM+ 20% FBS+ 1% Pen/Strep	1500
SR	lymphoma	NCI-Frederick cancer DCTD tumor/cell line repository	human		RPMI 1640+ 10%FBS+ 2mM Glutamine+ 1% Pen/Strep	8000
IGR-OV1	ovarian adenocarcinoma	NCI-Frederick cancer DCTD tumor/cell line repository	human		RPMI 1640+ 10%FBS+ 2mM Glutamine+ 1% Pen/Strep	1000

b

Drug Name	Mechanism/Targets	Drug Class	Solvent	Supplier	Supplier Ref	C1 (nM)	C2 (nM)	C3 (nM)	C4 (nM)	C5 (nM)	C6 (nM)	C7 (nM)	C8 (nM)
Axitinib	VEGFR, PDGFR, KIT inhibitor	B. Kinase inhibitor	DMSO	LC Laboratories	A-1107	0	1	3	10	30	100	300	1000
Bortezomib	Proteasome inhibitor (26S subunit)	A. Conv. Chemo	DMSO	ChemieTek	CT-BZ001	0	0,1	0,3	1	3	10	30	100
Cladribine	Antimetabolite; Purine analog	A. Conv. Chemo	DMSO	Medchem Express	HY-13599	0	1	3	10	30	100	300	1000
Crizotinib	ALK, c-Met inhibitor	B. Kinase inhibitor	DMSO	Selleck	S1068	0	0,1	0,3	1	3	10	30	100
Dactinomycin	RNA and DNA synthesis inhibitor	A. Conv. Chemo	DMSO	Medchem Express	HY-17559	0	1	3	10	30	100	300	1000
Erlotinib	EGFR inhibitor	B. Kinase inhibitor	DMSO	Medchem Express	HY-50896	0	10	30	100	300	1000	3000	10000
Everolimus	binds FKBP12, causes inhibition of mTORC1	C. Rapalog	DMSO	LC Laboratories	E-4040	0	0,1	0,3	1	3	10	30	100
Exemestane	Aromatase inhibitor	F. Hormone therapy	DMSO	Medchem Express	HY-13632	0	10	30	100	300	1000	3000	10000
Fulvestrant	Estrogen receptor antagonist	F. Hormone therapy	DMSO	Selleck	S1191	0	0,1	0,3	1	3	10	30	100
Gefitinib	EGFR inhibitor	B. Kinase inhibitor	DMSO	LC Laboratories	G-4408	0	1	3	10	30	100	300	1000
Lenalidomide	Immunomodulatory	D. Immunomodulatory	DMSO	LC Laboratories	L-5499	0	10	30	100	300	1000	3000	10000
Lomustine	Alkylating nitrosourea compound	A. Conv. Chemo	DMSO	Selleck	S2061	0	1	3	10	30	100	300	1000
Megestrol acetate	Progestogen	F. Hormone therapy	DMSO	Medchem Express	HY-13676	0	1	3	10	30	100	300	1000
Oxaliplatin	Platinum-based antineoplastic agent	A. Conv. Chemo	AQ	Selleck	S1001-3	0	1	3	10	30	100	300	1000
Romidepsin	HDAC inhibitor	E. Differentiating/ epigenetic modifier	DMSO	Medchem Express	HY-15149	0	0,3	1	3	10	30	100	300
Teniposide	Topoisomerase II inhibitor	A. Conv. Chemo	DMSO	Medchem Express	HY-13761	0	1	3	10	30	100	300	1000
Thioguanine	Antimetabolite; Purine analog	A. Conv. Chemo	DMSO	Medchem Express	HY-13765	0	1	3	10	30	100	300	1000
Vandetanib	VEGFR,EGFR, RET inhibitor	B. Kinase inhibitor	DMSO	LC Laboratories	V-9402	0	1	3	10	30	100	300	1000
Vismodegib	Smothened (Hh) inhibitor	X. Other	DMSO	LC Laboratories	V-4050	0	10	30	100	300	1000	3000	10000

c

Cell line: MALME-3M

Drug1	Drug2
Romidepsin	Vismodegib
Gefitinib	Vismodegib
Everolimus	Romidepsin
Cladribine	Romidepsin
Oxaliplatin	Romidepsin
Dactinomycin	Romidepsin

Cell line: SR

Drug1	Drug2
Bortezomib	Crizotinib
Lenalidomide	Teniposide
Crizotinib	Fulvestrant
Megestrol acetate	Thioguanine
Gefitinib	Lomustine

Cell line: HS-578T

Drug1	Drug2
Vismodegib	Romidepsin
Romidepsin	Everolimus

Cell line: IGR-OV1

Drug1	Drug2
Erlotinib	Axitinib
Lomustine	Gefitinib
Vandetanib	Exemestane

Table 4: Supplementary information on cell lines (a) and drugs (b) tested in experimental validation. (c) List of drug combinations tested in experimental validation, selected based on comboFM-5 predictions for specific cell lines.

Drug 1	Drug 2	Cell line	Tissue type	Trial numbers	Trial indication
Crizotinib	Fulvestrant	SR	LEUKEMIA	NCT03620643	Breast cancer
Everolimus	Fulvestrant	MCF7	BREAST	NCT01797120; NCT02753686	Breast cancer
Bortezomib	Romidepsin	SK-MEL-2	MELANOMA	NCT00963274	Chronic Lymphocytic Leukemia/Small Lymphocytic Lymphoma
Melphalan	Romidepsin	MALME-3M	MELANOMA	NCT01908777	T Cell Non-Hodgkin Lymphoma
Oxaliplatin	Romidepsin	MALME-3M	MELANOMA	NCT02181218	Relapsed/Refractory Aggressive Lymphomas
Gefitinib	Oxaliplatin	MDA-MB-468	BREAST	NCT00026299	Colorectal cancer

Table 5: Examples of monotherapy and combinatorial therapies identified by comboFM-5 in NCI-ALMANAC data that are being tested in clinical trials.

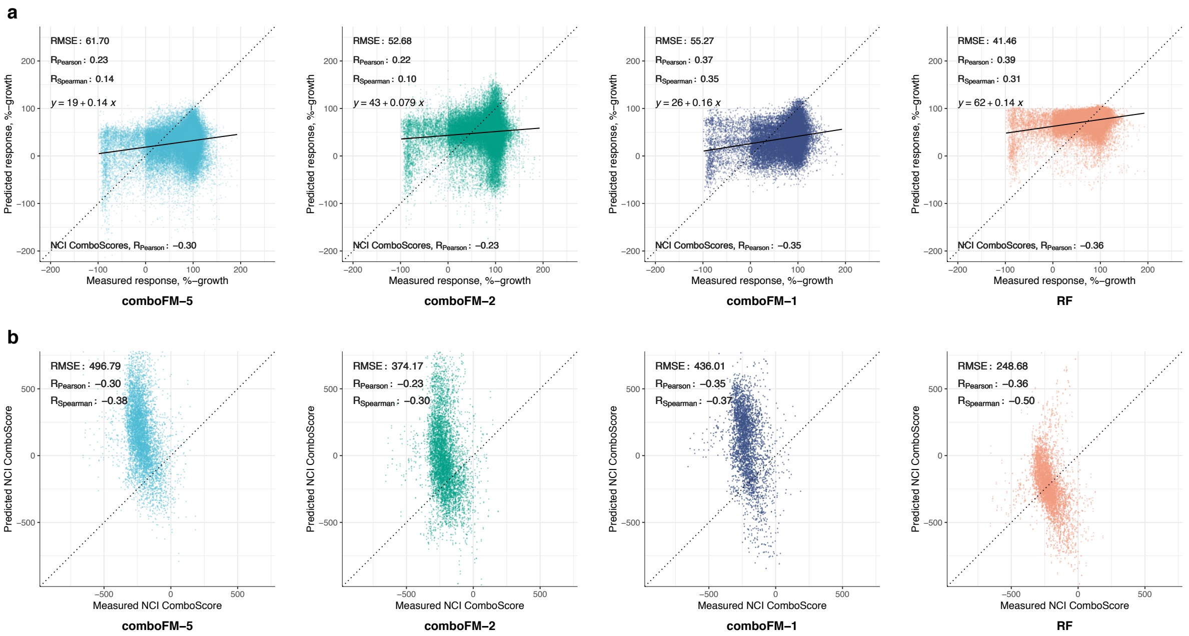

Figure 13: Root mean squared error (RMSE), Pearson correlation (R_{Pearson}), Spearman correlation (R_{Spearman}) and trend line equation of 1st (comboFM-1), 2nd (comboFM-2) and 5th order comboFM (comboFM-5) and random forest (RF) in a scenario where neither drug in a combination has been observed in any combination in the training set nor has the monotherapy responses of the drugs in the combination been observed, on a subset of the data consisting of 28 620 drug-dose responses as required by the split into the cross-validation folds. a) Scatter plots between the measured and predicted dose-responses. b) Scatter plots between measured and predicted NCI ComboScores computed based on predicted dose-response matrices. Trend line and its equation are shown for each scatter plot.
