# Supplementary material for: Leveraging multi-way interactions for systematic prediction of pre-clinical drug combination effects
Source: Nat Commun. 2020 Dec 1;11:6136. doi: 10.1038/s41467-020-19950-z (PMC7708835; doi:10.1038/s41467-020-19950-z)
Supplement: Supplementary file 3 — Reporting Summary [file 41467_2020_19950_MOESM3_ESM.pdf]

nature research

Corresponding author(s): Juho Rousu, Tero Aittokallio

Last updated by author(s): Nov 2, 2020

Reporting Summary

Nature Research wishes to improve the reproducibility of the work that we publish. This form provides structure for consistency and transparency in reporting. For further information on Nature Research policies, see Authors & Referees and the Editorial Policy Checklist .

Statistics

For all statistical analyses, confirm that the following items are present in the figure legend, table legend, main text, or Methods section.

n/a	Confirmed	
☐	☒	The exact sample size (*n*) for each experimental group/condition, given as a discrete number and unit of measurement
☐	☒	A statement on whether measurements were taken from distinct samples or whether the same sample was measured repeatedly
☐	☒	The statistical test(s) used AND whether they are one- or two-sided<br>*Only common tests should be described solely by name; describe more complex techniques in the Methods section.*
☐	☒	A description of all covariates tested
☐	☒	A description of any assumptions or corrections, such as tests of normality and adjustment for multiple comparisons
☐	☒	A full description of the statistical parameters including central tendency (e.g. means) or other basic estimates (e.g. regression coefficient) AND variation (e.g. standard deviation) or associated estimates of uncertainty (e.g. confidence intervals)
☐	☒	For null hypothesis testing, the test statistic (e.g. *F*, *t*, *r*) with confidence intervals, effect sizes, degrees of freedom and *P* value noted<br>*Give P values as exact values whenever suitable.*
☒	☐	For Bayesian analysis, information on the choice of priors and Markov chain Monte Carlo settings
☒	☐	For hierarchical and complex designs, identification of the appropriate level for tests and full reporting of outcomes
☐	☒	Estimates of effect sizes (e.g. Cohen's *d*, Pearson's *r*), indicating how they were calculated

Our web collection on statistics for biologists contains articles on many of the points above.

Software and code

Policy information about availability of computer code

Data collection	Code is available on Github: https://github.com/aalto-ics-kepaco/comboFM (DOI: https://doi.org/10.5281/zenodo.4129688).
Data analysis	Code is available on Github: https://github.com/aalto-ics-kepaco/comboFM (DOI: https://doi.org/10.5281/zenodo.4129688).

For manuscripts utilizing custom algorithms or software that are central to the research but not yet described in published literature, software must be made available to editors/reviewers. We strongly encourage code deposition in a community repository (e.g. GitHub). See the Nature Research guidelines for submitting code & software for further information.

Data

Policy information about availability of data

All manuscripts must include a data availability statement. This statement should provide the following information, where applicable:

- Accession codes, unique identifiers, or web links for publicly available datasets
- A list of figures that have associated raw data
- A description of any restrictions on data availability

The NCI-ALMANAC dataset is publicly available from National Cancer Institute (NCI) at https://wiki.nci.nih.gov/display/NCIDTPdata/NCI-ALMANAC.
The preprocessed data used in the computational experiments and in-house drug combination testing data for validating comboFM predictions are available at https://doi.org/10.5281/zenodo.4135059. Source data underlying the figures and display items are provided at https://doi.org/10.5281/zenodo.4135059 subdirectory source_data.

Field-specific reporting

Please select the one below that is the best fit for your research. If you are not sure, read the appropriate sections before making your selection.

- [x] Life sciences
- [ ] Behavioural & social sciences
- [ ] Ecological, evolutionary & environmental sciences

For a reference copy of the document with all sections, see nature.com/documents/nr-reporting-summary-flat.pdf

Life sciences study design

All studies must disclose on these points even when the disclosure is negative.

Sample size	The following sample sizes were used for the statistical tests: N=666360 (number of distinct dose-response matrix entries) for statistical tests on predicted dose-response values and N=74040 (number of distinct dose-response matrices) for prediction of synergy scores. The sample sizes are in the regime generally considered as 'large' for the statistical tests used.
Data exclusions	As a part of the preprocessing of the data available on the NCI-ALMANAC website (https://wiki.nci.nih.gov/display/NCIDTPdata/NCI-ALMANAC), a median across studies (experiment IDs) was taken and a subset was selected by randomly sampling 50 drugs from the original set of drugs, resulting in a dataset consisting of 617 distinct combinations tested in various concentration pairs across all the 60 cell lines,all cell lines were selected. Otherwise, no data was excluded.
Replication	The code for running the experiments is available on https://github.com/aalto-ics-kepaco/comboFM (DOI: https://doi.org/10.5281/zenodo.4135059) and the data is available on https://doi.org/10.5281/zenodo.4135059, hence allowing one to replicate and reproduce the findings.
Randomization	The dataset used in the experiments is based on a random sample. Cross-validation folds used to evaluate the computational performance of the method were randomly assigned.
Blinding	Blinding is not applicable since the development dataset contains full dose-response matrices of all drug combinations against each cell line and the nested cross-validation setup evaluates all of the combinations in unbiased manner.

Reporting for specific materials, systems and methods

We require information from authors about some types of materials, experimental systems and methods used in many studies. Here, indicate whether each material, system or method listed is relevant to your study. If you are not sure if a list item applies to your research, read the appropriate section before selecting a response.

Materials & experimental systems

n/a	Involved in the study	
x		Antibodies
	x	Eukaryotic cell lines
x		Palaeontology
x		Animals and other organisms
x		Human research participants
x		Clinical data

Methods

n/a	Involved in the study	
x		ChIP-seq
x		Flow cytometry
x		MRI-based neuroimaging

Eukaryotic cell lines

Policy information about cell lines

Cell line source(s)	Hs-578T & Malme-3M: ATCC and SR & IGR-OV1: NCI-Frederick DCTD tumor/cell lines repository
Authentication	Hs-578T & Mmalme-3M cell lines were authenticated using Promega GenePrint 10 System. Early passages of SR & IGR-OV1 cell lines on purchase were used, hence not re-authenticated.
Mycoplasma contamination	All the cell lines were tested negative for mycoplasma. The test was based on the method described by Choppa et al. and was performed as a service by the sample management laboratory of THL Biobank, Helsinki, Finland.
Commonly misidentified lines (See ICLAC register)	None used.
